# Phenotypic variability to medication management: an update on fragile X syndrome

**DOI:** 10.1186/s40246-023-00507-2

**Published:** 2023-07-07

**Authors:** Nasser A. Elhawary, Imad A. AlJahdali, Iman S. Abumansour, Zohor A. Azher, Alaa H. Falemban, Wefaq M. Madani, Wafaa Alosaimi, Ghydda Alghamdi, Ikhlas A. Sindi

**Affiliations:** 1 Department of Medical Genetics, College of Medicine, Umm Al-Qura University, Mecca, 21955 Saudi Arabia; 2 Department of Community Medicine, College of Medicine, Umm Al-Qura University, Mecca, Saudi Arabia; 3 Department of Pharmacology and Toxicology, College of Medicine, Umm Al-Qura University, Mecca, 24382 Saudi Arabia; 4 Department of Hematology and Immunology, Faculty of Medicine, Umm Al-Qura University, Mecca, Saudi Arabia; 5 Department of Hematology, Maternity and Children Hospital, Mecca, Saudi Arabia; 6 Department of Biology, Faculty of Science, King Abdulaziz University, Jeddah, 21589 Saudi Arabia; 7 Preparatory Year Program, Batterjee Medical College, Jeddah, 21442 Saudi Arabia

**Keywords:** Fragile X syndrome (FXS), Clinical features, Variable expressivity, *FMR1* gene, CGG trinucleotide repeat, DNA methylation, CRISPR/Cas9, dCas9

## Abstract

This review discusses the discovery, epidemiology, pathophysiology, genetic etiology, molecular diagnosis, and medication-based management of fragile X syndrome (FXS). It also highlights the syndrome’s variable expressivity and common comorbid and overlapping conditions. FXS is an X-linked dominant disorder associated with a wide spectrum of clinical features, including but not limited to intellectual disability, autism spectrum disorder, language deficits, macroorchidism, seizures, and anxiety. Its prevalence in the general population is approximately 1 in 5000–7000 men and 1 in 4000–6000 women worldwide. FXS is associated with the *fragile X messenger ribonucleoprotein 1* (*FMR1*) gene located at locus Xq27.3 and encodes the fragile X messenger ribonucleoprotein (FMRP). Most individuals with FXS have an *FMR1* allele with > 200 CGG repeats (full mutation) and hypermethylation of the CpG island proximal to the repeats, which silences the gene’s promoter. Some individuals have mosaicism in the size of the CGG repeats or in hypermethylation of the CpG island, both produce some FMRP and give rise to milder cognitive and behavioral deficits than in non-mosaic individuals with FXS. As in several monogenic disorders, modifier genes influence the penetrance of *FMR1* mutations and FXS’s variable expressivity by regulating the pathophysiological mechanisms related to the syndrome’s behavioral features. Although there is no cure for FXS, prenatal molecular diagnostic testing is recommended to facilitate early diagnosis. Pharmacologic agents can reduce some behavioral features of FXS, and researchers are investigating whether gene editing can be used to demethylate the FMR1 promoter region to improve patient outcomes. Moreover, clustered regularly interspaced palindromic repeats (CRISPR)/Cas9 and developed nuclease defective Cas9 (dCas9) strategies have promised options of genome editing in gain-of-function mutations to rewrite new genetic information into a specified DNA site, are also being studied.

## Background

Fragile X syndrome (FXS, MIM 300624) is an X-linked dominant disorder affecting approximately 1 in 5000–7000 men and 1 in 4000–6000 women worldwide [1]. Currently, it is the second leading cause of intellectual disability (ID) (2.4% of all ID cases), surpassed only by Down syndrome, the leading cause of inherited ID, and the leading cause of ID in male individuals [1–3].

FXS is caused by a CGG trinucleotide repeat expansion in the *FMR1* (MIM 309550). The typical number of CGG repeats in the FMR1 gene ranges from 5 to 44. Individuals with > 200 CGG repeats are considered to have a full FMR1 mutation, also known as a fragile site [4]. More than 99% of individuals diagnosed with FXS have the full mutation [5]. The presence of 55–200 repeats is considered an *FMR1* premutation, which is associated with fragile X-associated tremor/ataxia syndrome (FXTAS, MIM 300623) [6, 7] and, in female individuals, with fragile X-associated premature ovarian insufficiency (FXPOI; MIM 311360) [8, 9]. Several reports have shown that AGG interruptions within the *FMR1* gene also contribute to FXS [10]. The number of AGG interruptions and the length of uninterrupted CGG repeats at the 3' end of *FMR1* have correlated with repeat instability during transmission from parent to a child [11]. Maternal alleles with no AGG interruptions confer the greatest risk for unstable transmission of the CGG repeats [11].

The *FMR1* gene encodes the fragile X messenger ribonucleoprotein (FMRP), commonly found in the brain and essential for normal cognitive development and female reproductive function. FMRP can bind to ribosomes and regulate the translation of many mRNAs in postsynaptic neurons, which is critical for neurological development and function [12–15]. It has also been shown to bind to multiple ion channels to regulate their activity [16].

In mammals, cytosine methylation frequently occurs in the linear DNA sequence where DNA methyltransferases (DNMT) add methyl groups to a cytosine adjacent to guanine in a 5'-3' direction (a CpG island). DNA demethylation causes the replacement of 5-methylcytosine (5mC) in a DNA sequence by cytosine (C). DNA methylation is a major epigenetic modification that regulates transcription [17], and hypermethylation of this CpG island silences transcription of the *FMR1* gene [18]. The full FXS mutation is associated with hypermethylation of a CpG island, proximal to the CGG repeat, in the promoter of the *FMR1* gene. As a consequence, FMRP is not produced, and the absence or low expression of FMRP leads to FXS [19].

FXS is molecularly diagnosed based on relevant X chromosome abnormalities and alterations in the *FMR1* gene and is clinically diagnosed based on a wide spectrum of the physical, central nervous system, and neuropsychiatric/developmental features. Most male individuals with FXS cannot perform basic activities of daily living, e.g., feeding, ambulating, toileting, maintaining personal hygiene/grooming, and dressing. Female patients are often more self-reliant but frequently exhibit learning difficulties [20]. Although there is no recognized cure for FXS, psychosocial interventions, educational interventions, and drug treatment can help manage some aspects of the disorder [21–23].

### The early history of FXS

FXS, also known as Martin–Bell syndrome, was first described in 1943 by Martin and Bell as a form of ID following an X-linked pattern of inheritance [24]. Martin and Bell suggested X-linked inheritance because they observed that male children were more severely affected than their female counterparts. A family case study also suggested that FXS impaired brain development (likely the prefrontal cortex) since most patients had speech difficulty and ID [24]. Lubs first reported a fragile site at Chromosome Xq27.3 that segregated with ID in the late 1960s [25]. The association between this fragile site (FRAXA) and X-linked ID was confirmed two decades later [4]. Around the same time as Lub's discovery, Escalante et al. [26] noted the association of macroorchidism with X-linked mental retardation. Molecular analysis of 105 simplexes and 18 multiplex families later revealed no association between FRAXA and autism, ruling out Xq27.3 as a candidate region for autism [27]. Gross et al., however, have reported that the distinctive behavioral phenotypes of FXS-linked synaptic plasticity are consistent with autism spectrum disorder (ASD), self-injurious and stereotypic behavior, aggression, anxiety, impulsivity, hyperactivity, and attention deficit [28].

## Epidemiology

In Europe and North America, the prevalence of the full FXS mutation estimates at approximately 1/5000 male and 1/4000–8000 female individuals in the general population [14, 29–31]. Differences in haplotype frequencies and founder effects among different racial and ethnic populations can also affect the prevalence of FXS [32]. Newborn screening of 36,124 boys in the United States identified the full mutation in every 1 in 5,161 of the boys [30]; similarly, screening of 24,449 neonates in Québec, Canada, identified the full mutation in 1 in 6,209 boys [31]. The prevalence of the full mutation in male individuals is higher in Hispanic countries such as Chile (6.7%) [33], Spain (6.4%) [34–36], and Colombia (4.82%) [37]. Among 574 developmentally disabled French individuals, the prevalence of FXS was 1.9% (11/574) overall and 2.5% (10/403) in male individuals; only one case of FXS was detected among the 171 girls tested (0.6%) [38]. In 504 mentally disabled Iranian patients, full *FMR1* mutations were found in 19 (15.3%) of 124 unrelated families and in 13 (3.4%) of 384 consanguineous families [39]. Zhang et al. [40] have found a lower prevalence of FXS in a large-scale screening of 51,661 Chinese newborns (1/9,371 in males and 1/2,943 in females) than in Caucasians [1, 14, 29]. In the same study, they also found 33 children cohort of 33 children diagnosed with developmental delay. Among 237 Thai boys with a developmental delay of unknown cause in Southern Asia, 16 (6.8%) were found to have a full *FMR1* mutation, and four were reported to have a premutation [41]. The prevalence of FXS in Indonesia ranged from 0.9% to 1.9% among the ID population and was higher (6.15%) among the ASD population [42]. In Malaysia, with 2108 children with developmental disabilities from mixed ethnicities, the FXS full mutation was reported as 70 (3.6%) in males and 3 (2.4%) in females [43]. The prevalence of FXS in Pakistan was estimated to be 19/1,000 children for severe ID and 65/1000 children for mild ID [44]. In Northern Africa, the prevalence of FXS was 7.6% in 200 Tunisian boys with ID [45].

A global meta-analysis found that the prevalence of the FMR1 premutation ranges from 1 in 250–813 male individuals to 1 in 110–270 female individuals [1], much higher than the prevalence of the full mutation. In one study of male newborn screening in Spain, the prevalence of the premutation (1 in 1,233 male infants) was about ten times higher than the prevalence of the full mutation (1 in 2,466 male infants) [34]. It is noteworthy that the prevalence of the premutation is highest in Colombia and Israel (1 in 100 female individuals) and lowest in Japan (1 in 1,674 female individuals) [46]. In Saudi Arabia, screening of 94 cases with undiagnosed mental retardation found an even higher prevalence of the premutation: 6.4 in 100 female individuals and 7.86 in 100 male individuals [47]. In Egyptian males with ID, autistic-like features, and behavioral difficulties (*n* = 92), Rafeat et al. [48] found a prevalence of 37%, 0.03%, and 0.07% in premutation, gray zone (45–55 CGG repeats), and full mutation, respectively.

### Clinical characteristics of FXS

**Fig. 1:**
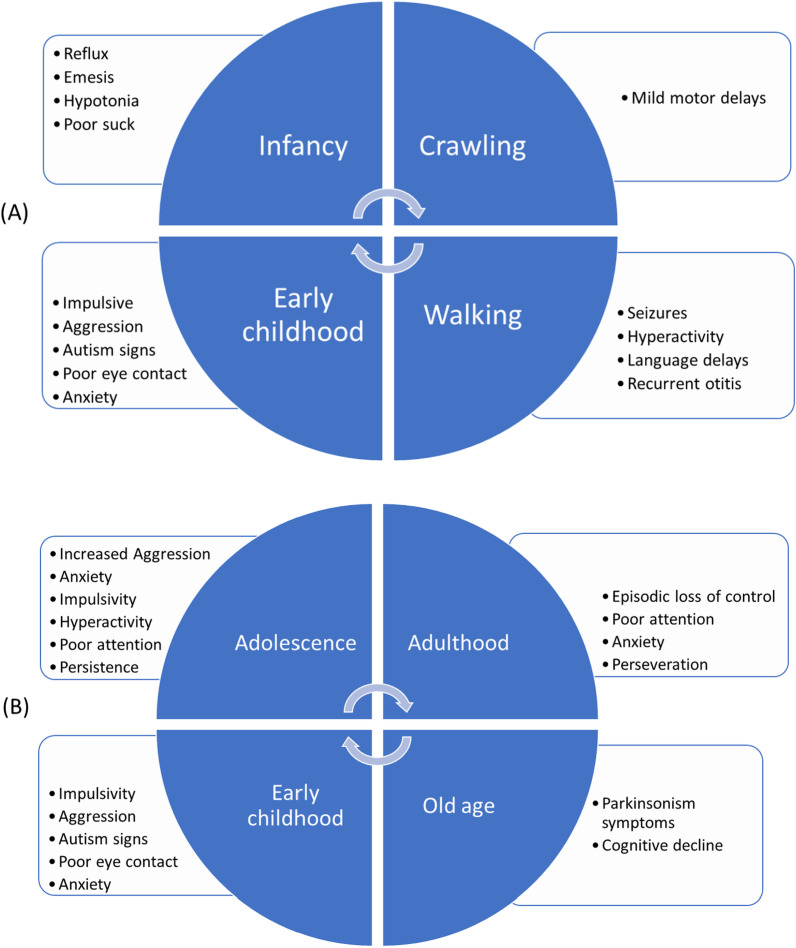
Clinical characteristics of fragile X syndrome **A** specific to infancy and early childhood and **B** spanning from infancy to old age. Adapted from [14]

At birth, the physical features associated with FXS are usually not apparent and affect children's height, weight, and head circumference within normal ranges [49]. Neonates show no clinical signs except for hypotonia, which is common among the general population [50]. Many of the major clinical characteristics of boys with FXS, summarized in Table 1 [51–54], become clearer during the first year of life, and diagnosis is often made around 2–3 years of life, particularly alongside the development of language delays [54, 55]. Figure 1 shows the most prominent clinical characteristics of the condition at each stage of life, from infancy to old age.

**Table 1:** Major clinical characteristics of boys with FXS

Category	Clinical characteristic	Prevalence (%)
Physical	Long/narrow face	83% more common in adults
	Macrocephaly	50–81%
	Prominent ears	72–78%
	Prominent jaws	80% in adults
	Flat feet	29–69%
	Macroorchidism	95% since adolescence
	Joint hypermobility	50–70% more common in boys
Central nervous system	EEG anomalies	74%
	Epilepsy	10–20%
	Brain MRI anomalies	Up to 50% of patients with neurologic morbidity
Neuropsychiatric/Developmental	Psychomotor delay	99%
	Intellectual disability	85%
	Aggressiveness	90%
	Attention problems	74–84%
	Anxiety	58–86%
	Hyperactivity	50–66%
	ASD	30–50%
	Sleep problems	30%
	ADHD	80%
	Depression	8–12%
Other	Strabismus	8–30%
	Nystagmus	5–13%
	Otitis	50–75% of children
	Gastrointestinal problems	30%
	Obesity/overweight	30–60%

Adapted from [51–54, 56]

*ASD* autism spectrum disorder, *ADHD* attention deficit hyperactivity disorder, *EEG* electroencephalogram, *MRI* magnetic resonance imaging, *NA* not available

The clinical presentation of FXS varies, as some primary and secondary clinical characteristics are more common than others (Table 1). The presentation also differs between girls and boys. For example, 85% of male patients but only 60% of female patients have ID [49]. In males, the characteristic phenotype also includes post-pubertal macroorchidism (i.e., enlarged testes), a prominent lower jaw, a narrow-elongated face, and large anteverted ears [49, 57]. Notably, however, 25% of male adults diagnosed with FXS do not have the distinctive facial characteristics associated with the condition [52]. Both girls and boys diagnosed with FXS tend to have connective tissue anomalies that can lead to heart disease (e.g., mitral valve prolapses) and are atypically short but otherwise have a normal physical appearance. Overall, the clinical presentation of FXS is less observable and more variable in female individuals than in their male counterparts [58].

### Brain imaging abnormalities

Almost 74% of FXS patients have been shown to have electroencephalogram (EEG) abnormalities, such as focal spikes originating from several anatomic parts. However, 35% of children with FXS report remission of EEG abnormalities by age 7 or 8 [59]. Additionally, patients with FXS have abnormal brain MRI scans. Notable defects include elevated cortical complexity, increased whole lobar and cortical thickness volume, and diffuse atrophy [60]. The anomalies can be associated with an undeveloped spine, increased spine length and density, and reduced pruning. Moreover, patients with FXS are at high risk of developing mesial temporal sclerosis, enlarged fourth ventricles, and hippocampal complications [61]. MRI abnormalities are also negatively associated with cognitive performance among children diagnosed with FXS [62].

## Comorbid and overlapping conditions

Autism spectrum disorder (ASD) and attention deficit hyperactivity disorder (ADHD) are commonly comorbid conditions in individuals with FXS. Research has shown that about 60% of boys with the full FMR1 mutation are co-diagnosed with ASD or ADHD [14, 63–65]. Furthermore, compared to boys without FXS, boys diagnosed with FXS have higher rates of ASD, ADHD, and anxiety [66]. Additional research suggests that ASD symptoms appear during early childhood in 50–60% of male FXS patients and 20% of female FXS patients [67, 68]. Although FXS and ASD affect overlapping neurobiological pathways, clinical trials have shown that the two disorders do not respond equally to the same treatment, suggesting different molecular mechanisms underlying the shared symptomatology [69].

The co-occurrence of FXS with other genetic conditions has been occasionally reported; the full FMR1 mutation was described in a few Down syndrome cases [3] and in five female fetuses with mosaic Turner syndrome (45,X0/46,XX) [70]. Also, an autistic ID was found to be affected by FXS while having pathological MED12 variants of X-linked MED12 (MIM 300188) [71]. However, the autistic features in FXS are not due to a double genetic cause but can instead be attributed to the variable phenotypic spectrum of the syndrome. Although the co-occurrence of Duchenne muscular dystrophy (DMD) with non-contiguous genetic entities' mutational events is extremely rare, an unrecognized association of X-linked DMD (MIM 310200) with ASD was previously reported [72]. The dystrophin protein 71 (Dp71) is widely expressed in the brain, and learning difficulties and cognitive impairments are also prevalent in DMD patients [73, 74]. Moreover, the *MYT1L* gene (MIM 616521) is associated with obesity, epilepsy, speech delay, and aggression, and *PPP2R5D* (MIM 601646) gene is correlated with neurodevelopmental disorder. Therefore, Tabolacci et al. [75] have recently described three unrelated cases of FXS co-occurrence with DMD, *PPP2R5D*, and *MYT1L* genetic conditions.

## FMR1 gene interactions of *FMR1* and co-expressed genes

**Fig. 2:**
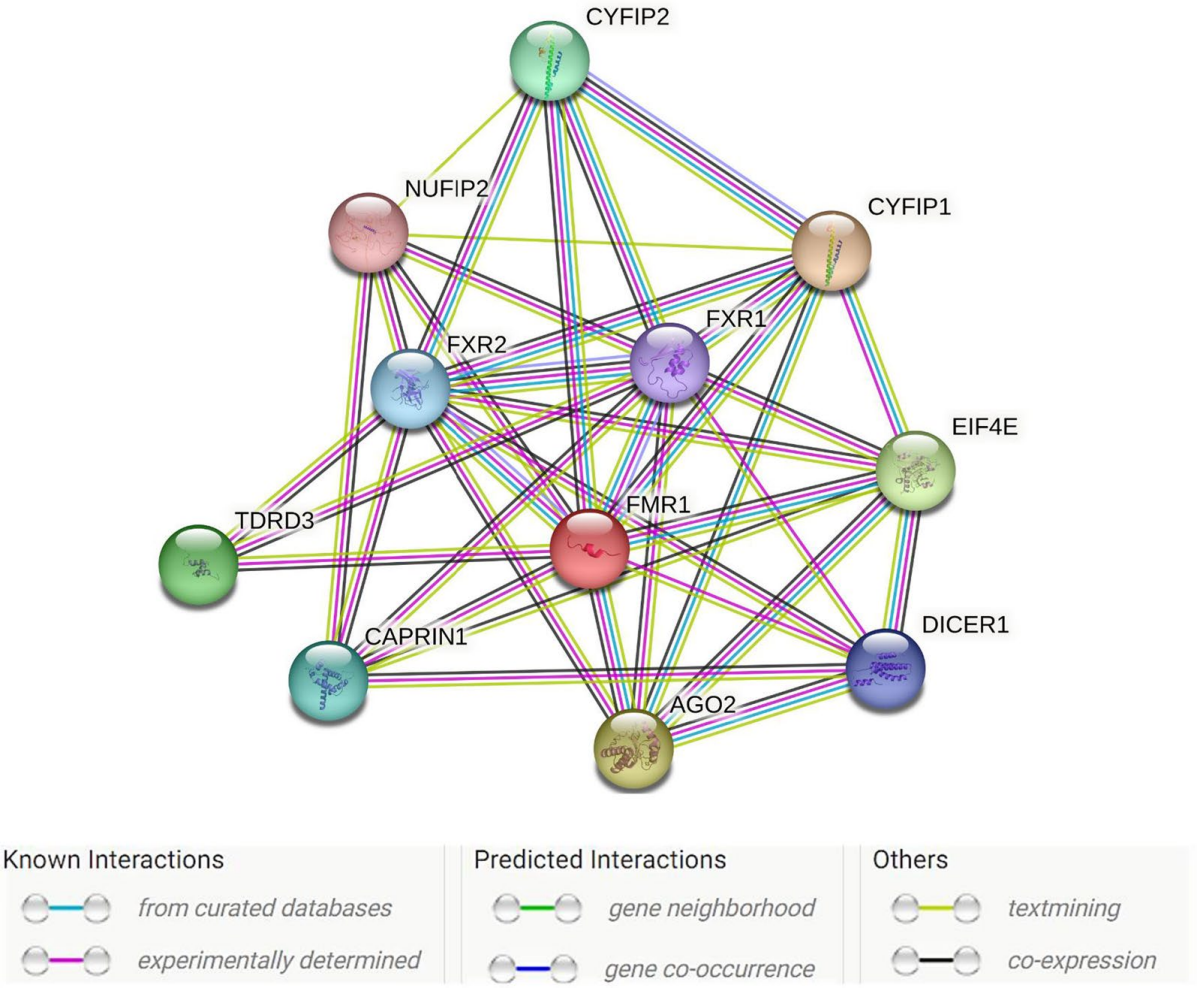
Protein–protein interactions predicted by STRING (https://string-db.org/). Strong interactions were predicted between *FMR1* and ten co-expressed proteins. Colored nodes (*n* = 11) represent proteins and the first shell of interactors (average node degree = 6.91). Edges represent specific and meaningful protein–protein associations (*n* = 38) (i.e., proteins jointly contribute to a shared function)

We analyzed the potential protein–protein interaction (PPI) network to predict functional interactions between proteins using the STRING database (https://string-db.org). Figure 2 presents the *FMR1* protein network interactions with STRING software. The FMRP protein network showed significantly more interactions among themselves (*P* value = 8.68e−10) than would be expected for random proteins of the same size and degree of distribution drawn from the genome. Such an enrichment indicates that the proteins are partially biologically connected.

## Functional enrichment analysis

Table 2 highlights the functional enrichment of FMRP and related proteins in biological processes, including the regulation of translation and modulation of synaptic transmission (GO:0099578) and regulation of gene silencing by miRNAs (GO:2000637), cellular components, including dendritic filopodium, and dendritic spine neck (GO:1902737/Dendritic spine neck), and cytoplasmic stress granule (GO:0010494). Furthermore, KEGG pathway analysis revealed the RNA transport (hsa03013), and protein domains revealed the fragile-X 1 protein core C-terminal (PF12235) (Table 2).

**Table 2:** Functional enrichment in FMR1 protein-coding gene loci network

GO-term	Function	Count in network	Strength	FDR
**Biological functions**				
GO:0099578	Regulation of translation at postsynapse, modulating synaptic transmission	2/3	3/07	0.0039
GO:0035087	siRNA loading onto RISC involved in RNA interference	2/4	2.95	0.0039
GO:0051388	Positive regulation of neurotrophin TRK receptor signaling pathway	2/7	2.71	0.0055
GO:2000637	Positive regulation of gene silencing by miRNA	3/24	2.35	0.00082
GO:2001022	Positive regulation of response to DNA damage stimulus	3/106	1.7	0.0096
**Cellular component (gene ontology)**				
GO:1902737	Dendritic filopodium	3/4	3.13	1.31e−6
GO:0044326	Dendritic spine neck	3/7	2.88	2.98e−6
GO:0005845	mRNA cap binding complex	4/11	2.81	4.16e−8
GO:0016442	RISC-loading complex	2/8	2.65	0.00073
GO:0010494	Cytoplasmic stress granule	4/73	1.99	9.34e−6
**KEGG pathways**				
hsa03013	RNA transport	5/160	1.74	5.96e−6
**Disease-gene associations (DISEASES)**				
DOID:14261	Fragile X syndrome	4/6	3.07	4.94e−8
**Protein domains (Pfam)**				
PF12235	Fragile X-related 1 protein core C-terminal	3/3	3.25	9.09e−6
PF05994	Cytoplasmic fragile-X interacting family	2/2	3.25	0.0017
PF00013	KH domain	3/39	2.14	0.0017

*FDR* false discovery rate, *GO* gene ontology, *KEGG* Kyoto Encyclopedia of Genes and Genomes

^a^ Number of proteins in the examined network/total number of proteins

^b^ Log_10_ (observed/expected), describing the extent of the enrichment effect

## Molecular and phenotypic variability

Mutations in the *FMR1* gene due to the CGG repetition can result in several conditions, e.g., ID, FXPOI, FXTAS, autism, Parkinson’s disease, developmental delays, other cognitive deficits, and even fragile X-associated neuropsychiatric disorders (FXAND) [53, 76, 77]. So, FXAND refers to the neuropsychiatric problems that typically occur at an earlier age than FXTAS. Hence, the *FMR1* premutation would exhibit variable expressivity and be associated with a wide spectrum of clinical phenotypes [78].

### Mosaicism

Mosaicism has been reported as a source of phenotypic variability in FXS patients of both sexes, with a higher frequency in male patients [78]. The prevalence of mosaicism in male FXS patients varies greatly, from 12 to 41% in the general population [79]. Notably, in individuals with FXS, mosaicism can be either in the size of the CGG repeat expansion or in hypermethylation of the CpG island [80]. A recent study has evaluated alterations in FMR1 function due to both types of mosaicism [78]. Because mosaic individuals with FXS produce some FMRP, they have milder cognitive and behavioral deficits than non-mosaic individuals with FXS [81, 82].

### Mosaicism of size

Mosaicism of size is described as the presence of both the full FMR1 mutation and the FMR1 premutation in some cells [79]. Approximately 50% of individuals with FXS are estimated to have this type of mosaicism [83]. FXS patients with mosaicism of size have higher IQ scores than those without mosaicism [84]. Although decreases in the number of CGG repeats (from full mutation to premutation and from premutation to normal size) are widely reported between generations, a retraction from full mutation to normal size appears to be sporadic [79, 85, 86]. In these cases, shortening of the CGG repeat expansion occurs post-zygotically due to the excision of many trinucleotides, giving rise to some alleles of normal size [86]. Baker et al. [87] reported that FXS patients with mosaicism of size had less aggressive behavior than FXS patients without this type of mosaicism. Mosaicism of size has also been associated with a higher risk of developing FXTAS [18].

### Mosaicism of hypermethylation

Epigenetic silencing of the *FMR1* gene in individuals with the full FMRI mutation is characterized by DNA methylation of the promoter region and modification of histones [88]. *FMR1* silencing takes place at about 11 weeks of gestation and seems related to histone H3 dimethylation, which is mediated by DNA-RNA duplex formation between the CGG repeat region of *FMR1* and its mRNA counterpart [89]. In mosaicism of hypermethylation, some cells exhibit hypermethylation, and others do not. In cells in which fully mutated or premutated alleles are not methylated, the *FMR1* gene is transcriptionally active and can be expressed [90–95]. In male individuals, the most frequent presentation of mosaicism is non-methylation of alleles with partial mutations and either methylation or non-methylation of alleles with full mutations (i.e., a combination of mosaicism of size and mosaicism of methylation) [14].

### Modifier genes

In several monogenic disorders, modifier genes have an important effect on the pathophysiological mechanisms regulating penetrance and expressivity [96]. A genetic variant can modify the phenotypic effects of other variants in many ways, including through epistasis and genetic interactions [97, 98]. Several studies have investigated modifier genes and their relationship with behavioral features of the FXS phenotype (e.g., epilepsy, aggression, autistic features) [99–103]. One study found that the Val66Met polymorphism in the *brain-derived neurotrophic factor *(*BDNF*) gene may lessen the epilepsy phenotype in FXS patients, as this polymorphism can affect cerebral anatomy [104] and fragile X-associated neuropsychiatric disorders (FXAND) [99–107]. Evidence has been conflicting on whether variations in genes such as *SLC6A4* (MIM 182138), *MAOA* (MIM 309850)*,* and *COMT* (MIM 116790) genes affect the severity of aggression, self-injury, and stereotypic behaviors in males with FXS [108]. However, Crawford et al. [103] recently reported that only *COMT*, and not *SLC6A4* or *MAOA*, can affect dopamine levels in the brain, contributing to variability in challenging and repetitive behaviors in male FXS patients.

## Molecular diagnostic testing

Although most parents notice some developmental delay during an affected child’s first year, FXS diagnosis may be delayed to 36 months. Diagnostic testing previously focused on karyotyping peripheral blood lymphocytes to determine if the X chromosome contained FRAXA [64, 109]. However, the test required advanced technical skills, and the results were challenging to interpret. Fluorescence in situ hybridization (FISH) later became the standard cytogenetic test for diagnosing FXS, given its high accuracy and reliability, but this has been replaced today by FMR1 DNA test. The American Academy of Pediatrics (https://www.aap.org/) recommends testing all individuals with ID, global developmental delay, or a family history of the full *FMR1* mutation or the *FMR1* premutation (Table 3).

**Table 3:** Clinical checklist to identify patients for molecular diagnostic testing

Clinical characteristic	Score if present	
	1	2
Soft, velvety skin on the palms with a redundancy of skin on the dorsum of the hand		X
Flat feet		X
Large and prominent ears		X
Plantar crease	X	
Macroorchidism (male patients after puberty)	X	
Family history of intellectual disability	X	
Autistic behavior	X	
Total	4	6

Each of the seven high-risk clinical characteristics is given 1 or 2 points (per the checklist) if present. The maximum total score is 10 for post-pubescent boys and 9 for girls and pre-pubescent boys. Molecular diagnostic testing should be considered in patients who score six or higher. Adapted from [114]

Many additional molecular diagnostic techniques have recently been developed for FXS [110]. Low-cost PCR using asymmetric oligonucleotide primers that anneal to the CGG motif in *FMR1* can screen individuals at high risk of FXS [35, 37, 111], and Southern blotting can be used as a confirmatory test [112]. Triplet repeat-primed PCR has also been recently introduced, allowing real-time magnification of the CGG repeats and full-length *FMR1* alleles [113], and DNA methylation analysis can be used to determine methylation patterns in some male patients [112]. To help determine which patients might benefit from molecular diagnostic testing for FXS, Lubala et al. [114] have developed an evidence-based clinical checklist for physicians (Table 3).

For prenatal molecular diagnosis, PCR-based *FMR1* testing is available using amniotic fluid samples (i.e., amniocytes) or chorionic villi samples. Current guidelines from the American College of Obstetricians and Gynecologists (ACOG) and the American College of Medical Genetics and Genomics (ACMG) encourage couples to have *FMR1* prenatal testing to facilitate early diagnosis. Notably, women with a personal history of isolated cognitive impairment, developmental delay, inexplicable ID, autism, elevated levels of the follicle-stimulating hormone after 40 years of age, idiopathic familial primary ovarian failure, isolated cerebellar ataxia accompanied with tremor, or FXS-related disorders are encouraged to have *FMR1* prenatal testing [13]. Importantly, there is also a need to perform AGG trinucleotide repeat genotyping [51], which can establish the magnitude of AGG interruptions within *FMR1* CGG repeats. This is especially important among women with a small premutation or borderline allele [52], as maternal alleles with no AGG repeats are at the greatest risk for *FMR1* CGG repeat instability and transmission [11]. Preconception is an ideal time for potential parents to request FXS testing or screening to make reasonable and evidence-based decisions about their reproductive health.

## Current and emerging therapeutic approaches

FXS currently has no cure, as no genetic manipulation, medical intervention, or medication has been shown to reverse the full impact of a lack of FMRP during fetal development [115]. However, pharmacological treatment aims to improve behavioral symptoms linked to FXS [115, 116], with sympatholytics, stimulants, antipsychotics, anxiolytics, and antidepressants being some of the most effective medications used for this purpose. Table 4 presents pharmacologic agents commonly used to treat some FXS phenotypes or have shown promise in recent clinical studies.

**Table 4:** Pharmacological agents commonly used to treat fragile X syndrome

Medication (Class of drugs)	Dose per day (Weight or age)	Target behaviors/issues	Side effects
Metformin (biguanide antidiabetic)	1000 mg (≤ 50 kg) 2000 mg (> 50 kg)	Social avoidance, low verbal and non-verbal IQ scores, aggression, macroorchidism	Nausea, diarrhea, headache, weight loss
Sertraline (SSRIs)	2.5–5.0 mg (2–6 y) 10–100 mg (> 6 y)	Anxiety, aggression, language development, social participation	Diarrhea, appetite loss, hyperhidrosis, tremor
Minocycline (tetracycline antibiotics)	2 mg (< 25 kg) 50 mg (25–45 kg) 100 mg (> 45 kg)	Mood dysregulation, anxiety	Nausea, diarrhea, headache, appetite loss, rash
Lovastatin (HMG-CoA reductase inhibitors) (statins)	10–40 mg	Anxiety, poor sleep, seizures	Weakness, gastro-intestinal disorders, muscle pain, dizziness, headache, irritability
Acamprosate (Psychiatry agents & GABA Analogs)	1332 mg (≤ 50 kg) 1998 mg (> 50 kg)	Dysfunctional chemical signaling in the brain	Irritability, anxiety, major depressive disorder, diarrhea
Cannabidiol (cannabinoids)	500–1000 mg	Anxiety, epilepsy, cognitive impairment	Hepatic abnormalities, diarrhea, fatigue, vomiting, somnolence

Adapted from [53, 69, 116–119]

*SSRIs* selective serotonin reuptake inhibitors, *HMG-CoA* 3-hydroxy-3-methyl glutaryl coenzyme, *GABA* Gamma-aminobutyric acid

### Metformin

*Metformin*, a biguanide antidiabetic agent, is a safe and effective therapy for type 2 diabetes and weight loss worldwide. Preclinical studies found metformin to be a modulator of the mGluR/mTORC1-ERK cascade in animal models of FXS [118, 120]. Metformin could correct social deficits, repetitive behaviors, macroorchidism, aberrant dendritic spine morphology, and exaggerated long-term depression of synaptic transmission in the adult FXS mice model (fmr1-/y mice) [120, 121]. Moreover, Dy and colleagues reported the first clinical data demonstrating metformin’s effectiveness in treating seven children with FXS for at least six months [118]. The FXS patients had improved weight and eating behaviors and experienced positive behavioral changes in irritability, social avoidance, and aggression [118].

### Sertraline

*Sertraline*, a selective serotonin reuptake inhibitor (SSRI), was the first antidepressant to treat anxiety in patients with FXS, including those as young as 2–3 years old starting in 2–3 years of life [69, 122]. SSRIs are described to stimulate neurogenesis, increase BDNF in FXS [123], and enhance dopamine levels in the striatum [124]. Both of these strengths can be very important for young children with FXS who have evidence of oxidative stress [125]. Metabolomic studies demonstrate the downregulation of the enzymes leading to serotonin production from tryptophan in the blood of patients with idiopathic ASD, including those with FXS [126, 127].

### Minocycline

*Minocycline*, a tetracycline antibiotic used to treat acne in adolescence, has been reported to decrease matrix metallopeptidase-9 (MMP9) level that is too high in FXS [128, 129]. MMP9 regulates synaptic formation (GSK3β, Arc, STEP, Map1B, αCaMKII), central nervous system development, and neural plasticity [130]. The cross between an MMP9 knockout mouse with an *fmr1* knockout mouse led to the rescue of the FXS phenotype in the offspring emphasizing the importance of the MMP9 pathway in the phenotype of FXS [127, 131].

### Lovastatin

*Lovastatin*, a 3-hydroxy-3-methylglutaryl-CoA (HMG-CoA) reductase inhibitor (also known as a statin), inhibits the RAS-MAPK-ERK1/2 activation pathway. In the FMR1 knockout mouse model, this has been shown to normalize the excess protein synthesis and prevent epileptogenesis, a functional consequence of increased protein synthesis in FXS [132]. In addition, they rescued the seizure phenotype in the live knockout mouse. These studies have encouraged clinical trials of lovastatin (10–40 mg/day) combined with a treatment of a parent-implemented language intervention in youth with FXS aged 10 to 17 years [133].

### Acamprosate

*Acamprosate* is a drug approved for maintaining abstinence in adults from alcohol. However, acamprosate has recently been focused on due to its potential pleiotropic effects impacting glutamate and GABA neurotransmission. A 10-week acamprosate clinical trial in 12 children with FXS ages 6 to 17 years showed improvements in social behavior and inattention/hyperactivity in 75% (9 children) of the study participants [134]. A multicenter controlled trial of acamprosate was carried out in individuals with FXS (http://www.clinicaltrials.gov; NCT01911455).

### Cannabidiol

*Cannabidiol (CBD)*, an herbal drug supplement extracted from cannabis plants, is mainly related to anxiety, cognition, movement disorders, and pain. In 2018, CBD was approved by the United States Food and Drug Administration to treat two epilepsy disorders. CBD represents a promising treatment to address comorbidities in FXS, e.g., epilepsy and cognitive impairment [135]. In humans, no clinical improvement with Huntington’s disease was shown [136], while clinical neuroprotection of CBD in general Parkinson’s was observed with no psychiatric comorbidity [137]. The transdermal gel of CBD was applied to children with FXS and showed efficacy in reducing anxiety and improving other behavioral measures [138]. Palumbo et al. [119] have recently reviewed the potential mechanisms for benefit from CBD treatment. Thus, the drug affects DNA methylation, serotonin 5HT-1A signal transduction, gamma-aminobutyric acid receptor signaling, and dopamine D2/D3 receptor signaling, which may help restore synaptic homeostasis in patients with FXS [119]. In many countries, CBD is legally sold at *marijuana* stores or online and is thus available for clinical use.

### Other therapeutic approaches

Several supplements, additional medications, and a gene editing approach have also been used or proposed to be used as a treatment for various clinical and molecular characteristics of FXS. *Folic acid* is an important micronutrient that facilitates the hydroxylation and methylation of neurotransmitters. Its therapeutic effects include improved speech, language, and motor coordination among patients with FXS [139]. L-acetylcarnitine has been used alongside methylphenidate or mixed amphetamine salts to treat co-morbid ADHD in FXS patients [140]. Treatment of *fmr1*^*KO*^ mice with the metabotropic glutamate receptor (mGluR) antagonist ‘2-methyl-6-(phenylethynyl) pyridine’ resulted in suppression of the audiogenic seizure phenotype [141] and rescue of dendritic spine morphology in the *fmr1*^*KO*^ mouse [142]. Moreover, mGluR antagonists can target features of macroorchidism, hippocampus atrophy, protein synthesis, and dendritic spine morphology [143]. Despite their promise, mGluR antagonists are still experimental drugs that must be comprehensively investigated in clinical trials to establish their efficacy [144].

## CRISPR/Cas9 gene therapy

Epigenetic modifying drugs can only transiently and modestly induce FMR1 reactivation in the presence of the expanded CGG repeat. Thus, gene replacements in gene therapy approaches are the most suitable option for those disorders caused by loss-of-function (LoF) mutations. In contrast, the gene replacement approach is not an option in gain-of-function (GoF) mutations to reduce the gene expression of the mutant target genes [145, 146]. The recent discovery of clustered regularly interspaced short palindromic repeats (CRISPR)/Cas9 strategies [147–151] has been developed to correct disease-causing mutations mammalian genome of living organisms [89, 152–156].

CRISPR/Cas9 system has been applied for genome editing in both GoF and LoF mutations by inducing double-stranded DNA (dsDNA) at specific loci. Cas9 can inactivate alleles with GoF mutations by inserting indels at sites associated with a single guide RNA (sgRNA) to form double-stranded breaks [157–161]. Thus, CRISPR/Cas9 has been applied to efficiently and directly demethylate the FMR1 triplet expansions [113]. A great development of nuclease defective Cas9 (dCas9) has been implemented to allow its binding to target genomic DNA sequences, creating steric hindrance that prevents the activity of other DNA-binding proteins such as endogenous transcription factors and RNA polymerase II and therefore interfering with gene expression (CRISPR interference) [162]. Thus, dCas9 has been fused to the catalytic domain of DNMT3A [163, 164] and ten-eleven translocation (TET) proteins to methylate and demethylate DNA [165]. Finally, dCas9 fusion to an engineered reverse transcriptase makes it possible to rewrite new genetic information into a specified DNA site.

## Conclusion

This review discussed the discovery, epidemiology, pathophysiology, genetic etiology, molecular diagnosis, and medication-based management of FXS. It also highlights the molecular mechanisms underlying the syndrome’s variable expressivity and summarizes several emerging, promising therapeutic strategies. Importantly, the content of this review can inform future public health studies on FXS and provide clinicians with evidence-based information about FXS and its genetic and clinical implications for patients and their primary caregivers.

## Data Availability

The data sets analyzed during the current study are available from the corresponding author.

## Abbreviations

ACMG: American College of Medical Genetics and Genomics
ACOG: American College of Obstetricians and Gynecologists
ADHD: Attention Deficit Hyperactive Disorder
ADHD: Attention deficit hyperactivity disorder
ASD: Autism spectrum disorder
CBD: Cannabidiol
COMT: Catechol-o-methyltransferase
CRISPR: Clustered regularly interspaced short palindromic repeats
dCas9: Nuclear defective Cas9
DMD: Duchenne muscular dystrophy
DNMT: DNA methyltransferase
EEG: Electroencephalogram
FDR: False discovery rate
FMR1: Fragile X mental retardation 1 gene
FMRP: Fragile X messenger ribonucleoprotein
Fragile X-A: FRAXA
FSH: Follicle-stimulating hormone
FXAND: Fragile X associated psychiatric disorders
FXPOI: Fragile X-associated premature ovarian insufficiency
FXS: Fragile X syndrome
FXTAS: Fragile X-associated tremor/ataxia syndrome
GO: Gene ontology
GoF: Gain-of-function
ID: Intellectual disability
KEGG: Kyoto Encyclopedia of Genes and Genomes
LoF: Loss of function
5-mC: 5-Methylcytosine
MAOA: Monoamine oxidase
mGluR: Metabotropic glutamate receptor
MRI: Magnetic resonance image
*MYT1L*: Myelin transcription factor 1-like
PPI: Protein–protein interaction
PPP2R5D: Protein phosphatase 2, regulatory subunit B (B56) delta
sgRNA: Single guide RNA
SLC6A4: Solute carrier family 6 (neurotransmitter transporter), member 4
TET: Ten-eleven translocation
